# Triple-layered security system: reliable and secured image communications over 5G and beyond networks

**DOI:** 10.1038/s41598-025-10022-0

**Published:** 2025-08-05

**Authors:** Tarek Srour, Mohsen A. M. El-Bendary, Mostafa Eltokhy, Atef E. Abouelazm

**Affiliations:** 1 Department of Electronics Technology, Faculty of Technology and Education, Helwan University, Cairo, Egypt; 2 Department of Electronics and Electrical Communications, Faculty of Electronic Engineering, Menoufia University, Menouf, Egypt

**Keywords:** 5G and beyond networks, Security, Loss-less steganography, Encryption techniques, Data hiding techniques, Layered-security adaptation, Environmental sciences, Chemical engineering, Electrical and electronic engineering

## Abstract

The vision of 5G and beyond networks is geared towards linking undersea, terrestrial, and space networks together. This vision involves transferring a massive amount of data with very high levels of security. This paper presents the proposed vision of 5G and beyond security to build a research gap of existing and related technique that lack the adaptation, boosting gradient and complexity analysis, through design and evaluate the adapted and graded security system. This paper focuses on designing a security approach to make up the lacks of existing techniques. The paper proposes a Loss-less Triple-level security system that secures the data without loss and high quality of extracted messages. The Triple-level security system consists of three combined/ merged security levels, the classified images are encrypted by two chaos-based tools, the encrypted images are embedded into another fake image or audio file using Singular Value Decomposition-Discrete Wavelet Transform (SVD-DWT) based techniques. Various simulation experiments have been executed to find the best integration between two-Dimensional (2D) Logistic map, SVD, and baker map techniques to ensure the integrity of plaintext and the transparency of the proposed approach. The result analysis of the various computerized experiments reveals that the time of algorithms are 2.13s for ciphering/hiding and 1.57s for deciphering/extracting. Hence, the time complexity of the proposed approach superiors the existing and related research works. The simulation results indicates a 100% perfect match between the original and decrypted. The performance evaluating of the proposed technique proves its robustness, low complexity and high reliability, where the original and extracted message similarity is 100%. The advanced wireless networks require reliable graded complexity security tools with various levels capabilities, therefore, the proposed adapted complexity/levels security system is suitable and applicable for 5G/beyond networks.

## Introduction

In the recent years, the world has been working on significant advancements in mobile wireless communication networks. We are living in a continuous evolution of generations of mobile wireless communication, starting from 1G, through 2G, 3G, and 4G, until the emergence of 5G/beyond of mobile wireless communication. The 5G networks come with a lot of benefits, like higher transmission rates with lower latency, better system performance and reliability, smaller terminal device size, and more energy-efficient device and network designs^1^. With the variations of applications and fields of these advanced networks, security techniques must be robust and adapted to provide the security requirements for different transferred data. 5G networks are intended to facilitate a greater variety of applications, including real-time closed-loop robotic control, large-scale Internet of Things (IoT), autonomous vehicles, Augmented and Virtual Reality (AR/VR). The five-generation (5G) system comprises three distinct technical features: ultra-Reliable Low-Latency Communications (uRLLC), massive Machine-Type Communications (mMTC), and enhanced-Mobile Broad-Band (eMBB)^2–4^.

Many researchers are interested in proposing suggestions for 5G/beyond system for mobile wireless communications. These suggestions expect that 5G and beyond will overcome the challenges faced by 5G during usage with various applications. The proposed expectations for 5G/beyond vision indicate that it will provide high technical standards for energy-efficient and spectrum-efficient new modes of transportation. It also indicates support for 5G/beyond to connect to a billion devices. These networks have higher frequency spectrum, faster speed, enhanced capacity, adapted error control, adapted security and lower latency. It is also expected to support new applications such as precision medicine, natural disaster prediction, and other various applications ^5^.

Several research papers have been concerned about the security developing of 5G/beyond networks, as it is one of the fundamental pillars that must be highly efficient and adapted. High-level security must be provided for data on the network, given the expected immense amount of devices and data on the network, according to the vision of 5G/beyond networks. Based on this vision, the security of 5G/beyond networks will face many challenges that must be overcome by using highly efficient security techniques. One of the challenges facing the security of 5G and beyond network is the programming and intelligence of the network, which leads to the emergence of new threats in these networks target the artificial intelligence training process. Strong security solutions are essential to deal with emerging threats related to artificial intelligence, such as backdoor embedding and poisoning of training data in federated learning. Also, 5G network lack a global standard to enhance security strategies in various scenarios to meet diverse security requirements and crucial applications, while reducing overall costs. For example, when the remaining battery power of the device decreases, the complexity of the security systems used must be adjusted to provide longer device operation time. Basically, this adjustment process requires adaptive, boost-gradient security systems with a various layered. With the increasing heterogeneity, dynamics, and complexity in 5G and beyond network, security must be adaptively customizable for different services, power conditions, and other variable characteristics over time^6,7^.

There is numerous security techniques have been presented for securing 5G/beyond networks^8–14^. These research papers have suffer from some weakness points and missed essential factors such as the adaptively, flexibility and power efficiency of the proposed security techniques^15–18^. In the brief, we describe the weaknesses points of these current works to build the research gaps as described in section “Related works”.In this presented paper, we focus on additional features of the proposed security techniques of 5Gbeyond networks compared to the existing and recent research works..

Our paper proposes a Triple-level security system that helps address some of the security challenges of 5G/beyond networks. The Triple-level security system consists of integrating multi-encryption techniques and loss-less data hiding techniques. So, it provides a high level of security of the transferred data on the network, the privacy and confidentiality have been provided. Additionally, it can be considered an efficient in case of power source limitations of the devices on the network, as it can be one of the energy-saving security systems. Therefore, the proposed system may contribute to solving one of the main challenges facing the security of 5G/beyond networks.

### The main contributions

In this sub-section, the main contributions of our presented research paper have been discussed. We present a wide comprehensive existing technique and related research works survey for the 5G/beyond. Also, the presented research paper provides method to build the research gaps based on studying the existing related works for capturing the weakness points of these techniques. The most of related works missed the main required features in the 5G/beyond security approaches, which are the adaptively behavior for achieving the high securing link,optimizing the power efficiency and flexible layered algorithms. These factors have been considered in our pesented proposed work. The proposed combined Triple-security algorithm has been constructed based on chaos mapping-multi stages and loss-less steganographic techniques to achieve several features, such as the highest security level for the classified plaintext, enhanced the power efficiency due to the multi-levels, and adaptively algorithm based on the resources and classification level. The proposed security algorithm provides the optional multi-layered security without raising the complexity of the algorithm, as clarified in the operation mechanism of the proposed approach. The high flexibility of the proposed security algorithm has been achieved, due to its interaction with the various conditions based on the type of plaintext, the classification degree, the available energy ‘battery level’, and the environment of the communications.

The existing techniques suffer from some of the limitations and drawbacks. The main limitation of the real-time applications is the longer consumed time of cryptographic algorithms in the encryption and decryption processes. Also, the several recent related research works have missed considering of the time complexity in the evaluation of their proposed algorithms. Also, the real-time applications of 5G/beyond networks have been considered within the mechanism operation of the proposed Triple algorithm through limiting the number of round/level/layer security based on the applications and type of information.

Our main contributions can be listed as in the following:

1. Building research gap due to the most of the presented related missed the essential and vital features/properties of the 5G and beyond security tools which are the adaptively, power efficiency, the computational complexity and the levels grading-under the pre-conditions.
2. Our research paper presents a vision of 5G/beyond security techniques, that has been represented through proposing adapted, boosting-gradient and graded complexity Triple-layered security system.
3. The hybridizing behavior plus effective design for achieving the expected properties of 5G and beyond security techniques.
4. The proposed security approach has been designed to be more flexible in the security level grading and number of embedded rounds to control the complexity and time of the processes of ciphering and deciphering stages.
5. Mixing two permutation-based methods to enhance the ability of the algorithm/approach for resisting the different attacks through 2-D Baker chaos mapping and detecting any tiny tampering in the ciphered file through the high sensitive 2-D Logistic chaos mapping. These two various 2-D chaos mapping tools have been separated/isolated by loss-less data hiding SVD-based steganography

The rest of this paper is organized as follows: Section “Related works”discusses related works. Section “The proposed security approach of 5G/beyond networks” discusses the proposed approach of 5G and beyond security. Section “The objective metrics: performance evaluations” presents the objective metrics are used for evaluating the proposed technique. Section “Computer simulation and result discussion” presents the performance evaluating experiments the proposed technique revealed its robustness and high reliability. . Section “Conclusion” presents the conclusion of our proposed technique.

## Related works

The existing security techniques and the recent published related research works have been considered in this section to analyze these security approaches compared with the proposed security system in our research paper. From this survey, it is clarified that the most of the presented related missed the essential and vital features/properties of the 5G/beyond security tools which are the adaptively, power efficiency, the computational complexity and the levels grading-under the pre- determined conditions. The last property concerns the conditions of the available resources of the mobile nodes, the probably threats, indoor/outdoor applications and others conditions based on the applications.

The second point has been concluded form the survey is that the most of presented related works have been proposed based on the hybridization concept to raise the security level using classic tools such as the ECC, AES and chaotic mapping or merging with the steganography data hiding technique. Our proposed security approach tries to build research gap through utilizing the classic security tools in hybridizing behavior but presenting effective design for achieving the expected properties of 5G/beyond security techniques. The proposed security approach has been designed to be flexible in amount of time complexity for the real-time applications.

Based on the related works survey, there are four basics categories of proposed techniques, which are the standalone/hybrid cryptographic approach, data hiding-steganographic, the hybridization of cryptography and data hiding-steganographic-data transforms and cryptographic-data transforms approaches. The characteristics of these related research works and their pros and cons have been tabulated in Table 1 as clarified in the last of this discussion with respect to the category, the utilized techniques, the complexity, advantages and drawbacks and publishing year. The recent related research works have been discussed as in the following.

**Table 1:** Comparison of recent related works with respect to the various categories, metrics and complexity.

Related existing techniques Ref	Classification of techniques	Implemented techniques	Complexity degree stengths/weakness	Real-time applicability	Type of data	Metrics tools
Ref. ^19^	Encryption techniques: Standalone & Combined Multi-Encryption tools	2-D Logistic encryption with the Secure Hash Algorithm (SHA)-256 protocol	Moderate/ Multi-Security/High Complexity	Un-Considered	Images	Histogram,Correlation&Entropy
Ref. ^20^		DNA and CNN	Simple L1-Security/Low Complexity	Considered	Images	NPCR,Histogram,Correlation&Entropy
Ref. ^21^		Chaotic map	Simple low-Security/low Complexity	Considered	Images, Audio&Waves	PSNR,Correlation, Entropy & Time
Ref. ^22^		RSA, 2D-PSNCM and Baker map	High Multi-Security/High Complexity	Un-Considered	Images	Correlation, Histogram, MSE,PSNR & SSIM
Ref. ^23^		Hybrid chaotic maps, Elliptic Curve Cryptography and genetic algorithm	High Multi-Security/High Complexity	Un-Considered	Images	Histogram, Entropy, NPCR, UACI, PSNR &Correlation
Ref. ^24^		Chaotic maps	Medium Multi-Security/Medium Complexity	Considered	Images	Entropy, Correlation,PSNR,MSE& Histogram
Ref. ^25^		AES and RSA	Moderate Multi-Security/High Complexity	Considered	Medical Data	Total cost, Distance, Throughput & Time
Ref. ^26^		ECC, AES and RSA	High Multi-Security/High Complexity	Un- Considered	Medical Data	Time &Energy Consumption
Ref. ^27^	Data Hidingtechnique	Discrete tchebichef transform (DTT)	Simple Low-Security/Low Complexity	Considered	Images & Binary data	PSNR, SSIM & NC
Ref. ^28^		2D-LASM and QQIM	Moderate Multi-Security/High Complexity	Considered	Images	MSE, PSNR, SSIM, &NC
Ref. ^29^	Merged techniques (Encryption, Steganography & Data transform)	Elliptic-curve cryptography (ECC) and Hadamard transform	High Multi-Security/High Complexity	Un-Considered	Images & Binary data	PSNR,SSIM, NC & BER
Ref. ^30^		DWT & SVD	Medium Multi-Security/Low Complexity	Considered	Images	PSNR, MSE, SSIM, UIQI & NC
Ref. ^31^		DWT, DCT and chaotic maps	High Multi-Security/High Complexity	Un-Considered	Audio	Correlation, SNR, BER, NSCR & UACI
Ref. ^32^		Additive Secret Sharing (ASS), and Adaptive Bit-Plane Prediction (ABPP)	Medium multi-security/high complexity	Considered	Images	ER, PSNR, MSE, SSIM, NPCR & UACI
Ref. ^33^		IWT and SVD	Simple L1-security/low complexity	Considered	Images	PSNR, MSE, SSIM& NCC
Ref. ^34^		DNA and DCT	Simple low-security/low complexity	Considered	Images	PSNR, SSIM, SSIM & NCC
Ref. ^35^		DWT and chaotic maps	Medium multi-security/high complexity	Considered	Audio	Correlation, PSNR, MSE &SSIM
Ref. ^36^		Scrambling algorithm and multi-dimensional feature fusion algorithm	Medium multi-security/high complexity	Considered	Images	BEQ
Ref. ^37^	Data Transform & Chaos-Based	DWT, DST, DCT & Chaos Mapping/Multi-S_keys_	Medium multi-security/high complexity	Considered	Audio	Cr, MSE, Spectral Distortion, LLR, Histogram
Ref. ^38^		Data transforms& baker Chaos Mapping	Simple multi-security/low complexity	Considered	Image	Cr, PSNR, MSE

An approach of medical images encrypting over 5G networks has been proposed in^19^. The approach presented the concept of combining a different cryptography techniques, it combined 2-D logistic encryption with the Secure Hash Algorithm (SHA)-256 protocol. In this approach, the SHA protocol is responsible for generating encryption keys. The 2-D logistic encrypts images based on permutation and diffusion logistics using the encryption keys. The proposed approach ensures secure transmission of medical data over 5G networks it is characterized by acceptable resistance to different types of attacks. However, it is sensitive to initial conditions. On the other hands, it missed enough discussing its applicability for the real-time medical application, on the other hand, it has a moderate computational complexity.

In^20^, the authors presented an approach to encrypting colored images based on Deoxyribonucleic Acid (DNA) and Convolutional Neural Network(CNN). The proposed approach relies on generating a set of public and private encryption keys using both the CNN and Pseudo-Random Number Generator (PRNG). These keys are combined using the intertwining logistic map and then applied with the rules of DNA encoding. The secret images are encoded using DNA. Both diffusion and bit-reversal are applied to the encrypted image to provide additional security level. The proposed approach is characterized by its suitability for real-time multimedia applications. The combination of multi-tools concept has been adopted in this research paper as clarified, it utilizes multi-secret keys to enhance the confidentiality of a medical colored image through chaos-mapping, DNA and CNN. Due to the merging of these three tools, the complexity has been raised above moderate level and there is no a sufficient analysis of its suitability/applicability in the real-time applications. The cryptography based on the chaos mapping has been adopted to secure the data over the 5G network in^21^. The proposed crypto-system relies on the chaotic mapping as a tool to execute the encryption process through pseudo-random numbers. The proposed system is characterized by its ability to encrypt different types of data, such as aimages, audio, and waves. This approach is characterized by a simple data retrieval process and short steps, hence its complexity is very limited compared to the presented security approaches in^19,20^.

In^22^, the authors used the RSA public key cryptosystem and two chaotic maps to provide a robust multi-layers image ciphering strategy and a modified authentication method for gray-scale and color images. To increase the degree of confusion and permutations of rows and columns are carried out using a 2DPiecewise Smooth Non-linear Chaotic Map (2D-PSNCM). Additionally, bitwise XOR is used to provide a complex degree of diffusion. A sufficient number of iterations of the Baker map are used to construct the pixel scrambling in order to reduce correlation and increase the security. The RSA public key cryptosystem for partially encrypted images is suggested as the modified digital signature for authenticity purposes. As clarified from the contents of the proposed cryptographic system in^22^, the complexity increases due to the merging of multi-encrypting techniques.

The hybridization of various cryptographic techniques for achieving high degree of security has been adopted in^23^ to provide multi-levels of image encryption. The proposed approach uses hybrid chaotic maps, elliptic curve cryptography and genetic algorithm for image encryption. In this approach, images go through three encryption phases: the first phase applies Lorenz chaotic map for confusion and logistic piecewise linear chaotic map for pixels diffusion of the secret images. The second phase applies the elliptic curve cryptography to encrypt the image. The third phase processes the images with genetic algorithm to obtain an image encrypted with three different encryption levels. The proposed approach in^23^ is a good choice for securing the transmission of images across many applications. In^24^, the authors presented an approach to improve image encryption based on chaotic maps. The authors used eight chaotic maps for image encryption performance. The proposed approach can be used to enhance image encryption relies on two different processes (confusion and diffusion) during encryption. One of these two processes alters the position of the data, while the other changes the value of the data, that is leading to robust image encryption. As cleared in^23,24^, the complexity of the proposed technique in^23^ is higher than the proposed cryptosystem in^24^ due to the multi-cryptographic tools combining. On the other hand, the presented technique in^24^ is suitable for the real-time applications.

Data securing approach has been presented in^25^, for the application of medical monitoring sensors. The proposed approach relies on the integration of different techniques for encrypting the data of the medical sensors. This integration of various techniques provided a high level of security for the data on the network, the proposed approach combines AES and Rivest–Shamir–Adleman (RSA) techniques. In^26^, the authors presented an approach to securing data for smart home healthcare application, through combining the ECC, AES and RSA techniques to secure the transferred data.. In^27^, authors presented an algorithm for a strong watermark to secure images on 5G networks. The proposed algorithm relies on Discrete Tchebichef Transform (DTT) and Direct Current (DC) component. In this algorithm, the color image is divided into the R, G, and B channels, while the watermark is transformed into a consecutive series of binary numbers. The algorithm combines parts of the image with the binary numbers representing the watermark. The image parts are then gathered again to produce the color image embedded with the watermark.

In^28^, the authors discussed an approach to improve the coding of color images and providing increased security rates. The approach relies on two-dimensional Logistic-adjusted-Sine map (2D-LASM)(2D-LASM) and QQIM for embedding the watermark in the color image, it is characterized by resistance to attacks, embedding capacity and security. In^29^, the authors proposed an algorithm for securing colored images on 5G networks using a colored watermark. The approach relies on Elliptic-curve cryptography (ECC) encryption algorithm based on public key cryptography and Hadamard transform. The proposed algorithm relies on dividing both the colored images and the colored watermark into three parts: R, G, and B. The parts of the image are divided into a set of blocks, while the parts of the watermark are converted into digital information. The watermark is integrated into its digital form within the image parts. The image is reconstructed to form a colored image with the embedded watermark. This proposed approach is characterized by acceptable resistance to various attacks and suitable for large data security application.

In^30^, the authors presented an approach to securing images utilizing the watermarking technique. The combination of the Discrete Wavelet Transform (DWT) and Singular Value Decomposition (SVD) has been performed as an approach to image watermarking. The proposed method transforms the cover image and watermark from the spatial domain to the frequency domain using DWT. The SVD is applied to HH sub-band of both the cover image and the watermark, and then they are merged. Inverse Discrete Wavelet Transform (IDWT) is applied to the sub-bands of the cover image with the merged watermark to produce the watermarked image. The proposed approach is characterized by securing data in the frequency domain relying on the DWT, it has moderate resistance to salt and pepper attacks as clarified in the results analysis, it is also not suitable for binary watermarks.

In^31^, the authors discussed an approach to securing the audio data over wireless networks. The proposed approach relies on the integration of audio watermarking and encryption. In the watermarking integration stage with the audio, the approach depends on data transformation techniques such as DWT and Discrete Cosine Transform (DCT). In the audio encryption stage, the approach relies on mixing several chaotic encryption techniques such as tent map and Logistic map. The proposed approach is characterized by multiple levels of security for audio data, in addition to being a data lossless approach. In^32^, the authors presented an approach for encrypting and hiding data on 5G networks. The approach relied on Reversible data hiding in encrypted images (RDHEI), Additive Secret Sharing (ASS), and Adaptive Bit-Plane Prediction (ABPP) for encrypting and hiding the classified data. The cover image is encrypted in this approach and then divided into several parts. Each part of the encrypted image participates in hiding the secret data. The parts of the image are then combined again to produce the encrypted cover image, which contains the hidden secret data.

In^33^, the authors presented an approach to securing the medical images over 5G networks based on integer wavelet transform(IWT) and SVD tools. The approach relies on integrating a watermark into the medical images to provide a high level of security. In the proposed approach, the medical images are transformed from the spatial domain to the frequency domain using IWT. The sub-bands of the medical image, HL and LH, are applied to SVD for compression and merged with the watermark. Inverse integer wavelet transform (IIWT) is applied to the medical image sub-bands to obtain a watermarked medical image. The embedded process has been performed in frequency domain to raise the security level.

The watermarking technique has been considered for image securing on 5G networks in^34^. The approach relies on encrypting the watermark using chaotic and DNA techniques. In this approach, colored images are divided into two channels, each channel is transformed into the frequency domain using DCT. The encrypted watermark is embedded in each channel, each channel is retrieved back to the spatial domain using IDCT. The two channels are merged again to produce the watermarked colored image. Embedding the watermark in the frequency domain of images provided a high level of security characteristic of this approach. This proposed approach in^34^ is robust to both hybrid and singular attacks as proved in the results analysis.

In^35^, authors proposed a hybrid security approach that relies on both hiding and encryption techniques. The research utilized the chaotic map to encrypt images and hide them within an audio file. It also relied on using the data transform to convert between the spatial domain and the frequency domain. The approach depends on transforming the audio using the DWT to hide the image after encrypting process using the chaos mapping. Hence, the audio-watermarked is returned to the spatial domain using the IDWT, then, it is then sent over the wireless link of the communications channel. This proposed hybrid security method demonstrated high efficiency in securing images by relying on both hiding and encryption techniques.

In^36^, the authors presented an approach to secure secret data on beyond 5G networks, it relies on both encrypting and hiding of secret data to achieve a high level of security. The proposed approach depends on an information scrambling algorithm for encrypting secret data. Also it utilizes multi-dimensional feature fusion algorithm for hiding secret data. The secret data is encrypted using the encryption keys of the scrambling algorithm. The encrypted secret data is hidden in a host carrier using fusion algorithm for hiding. The proposed approach is characterized by providing a high level of security and acceptable performance against various types of attacks. However, it needs to achieve a better balance between the imperceptibility of the hidden information and its robustness.

The previous discussions of the recent related works have been tabulated in Table to brief their advantages and disadvantages. The main disadvantages of the related works can be included in three factors, first one is the degree of security robustness which is determined by the utilized techniques and the number of merged techniques. The second factor is the acceptance of the computational/time complexity considerations due to the employed techniques. The third factor is missing the adaptively property. So, Table 1 briefs the cons and pros of these presented related works based on the level of complexity (one of three levels, simple- medium and high) and the real-time applicability (as a metric of amount complexity acceptance) of the proposed cryptographic approach. The proposed crypto-system in our paper tries to build research gap to cover the three drawbacks of the related works, which can be briefed in these terms, robustness, flexible complexity and adaptively properties. The adaptive-Triple-Level security system has been designed and evaluated based on the hybridization and adaptation concepts to success in building the research gap of the 5G/beyond security approaches.

As clarified from the previous discussion, the four categories of the recent related works can be internally segmented to three sub-categories based on the complexity degree of the proposed cryptographic techniques as clarified in Table 1. On the other hand, the complexity degree of the cryptographic approach determines its suitability and degree of its applicability for the real-time applications. Based on the previous comprehensive discussion of the related works, the main categories of the proposed research works can be concluded as follows:

1. The most of related works adopted proposing a robust security approach based on hybridization a various security techniques such as the cryptographic/data hiding approaches^19–28^.
2. Also, the combining various cryptographic tools is adopted in a several research papers to establish a robust multi-encryption levels^29–36^.
3. The crypto-process in the time and frequency domain based on employing the data transform techniques has been presented in^37,38^.

From this survey, the presented recent related works suffer from same drawbacks, such as the weakness of proposed cryptographic technique, extensive complexity of some proposed approaches and missing of level-grading of security based on the pre-determined conditions. In our proposed crypto-system, we aim to solve these drawbacks through grading of level of the security to reduce and limit the complexity to be applicable for the real-time applications. Moreover, the proposed crypto-system provides unlimited security-layer according to the circumstances of transmission, nature of the processed information and the classification degree. As shown in section “Methodology of triple-level security system”, our proposed crypto-graphic adopts the merging concept due to employing two chaos mapping tools, data transform and loss-less data hiding-steganography-SVD based.

The SVD-based steganography provides lossless data hiding approach to hide original plaintext and ciphered version. The utilized two chaos maps are the baker and logistic maps, baker is high resistive of attacks and noise, while the logistic is high sensitive to any tiny tampering, it is applied in the proposed crypto-system scenario in the last stage of the algorithm.

## The proposed security approach of 5G/beyond networks

Due to the different environments in which 5G/beyond network will be used, it faces many challenges. One of the most important challenges is achieving the sufficient security requirements. These advanced network needs to provide a high level of security for the data transmitted. Our proposed vision relies on some traditional methods of securing data on communication networks within the concept of adaptation, hybrization and boosting-gradient. Traditional security methods are appropriate alternative to modern proposed methods for 5G/beyond network, such as Quantum Communication, block chain technologies and so on. Additionally, traditional data security methods may be the appropriate solution to overcome one of the challenges, which faces the sixth-generation network, which is energy consumption. Therefore, we present our vision based on traditional methods for securing data.

Our vision for the security of 5G/beyond relies on data encryption techniques and data hiding techniques. Our vision proposes an adaptive security system. The proposed security system is divided into one to Triple- levels of security. Our vision relies on the possibility of alternating between security levels based on the network application and the energy consumption levels within the network. If the data of the application being used does not require a high level of security, the system uses one level of security. This level can be either data hiding techniques only or data encryption techniques only. However, if the data of the application requires a high level of security, the system uses Triple-levels of security. The selected security layer is chosen based on various pre-determined conditions such as, the importance of the data, battery level, the energy consumption and etc. For example, in 2-level security system, the combination of data encryption techniques and data hiding techniques has been performed for achieving the required security stages. The data transmitted over the network passes through data encryption technique then through data hiding technique to become a message secured with two levels of security. While, in the situation of triple-level security system, this level is executed by combining of two different data encryption techniques with data hiding techniques. The data transmitted over the network passes through data encryption technology, then through data hiding technology, and then through another data encryption technology. In our approach, we rely on steganography technique for data hiding, represented by LSB and SVD techniques, while we rely on chaotic encryption techniques for data encryption, represented by the 2D Baker map and 2D Logistic map. Section “Singular value decomposition (SVD)” illustrates our proposed vision for the security of 5G/beyond network.

The proposed interactive-adaptive Triple-level security approach has been devoted and design to achieve the robustness, flexible complexity (Level-grading) and adaptively properties. It adopts the main two techniques, the decomposition process using singular Value Decomposition (SVD) and chaos cryptographic techniques utilizing two different chaos mapping. In the following a simple description of the utilized techniques:

### Singular value decomposition (SVD)

**Fig. 1:**
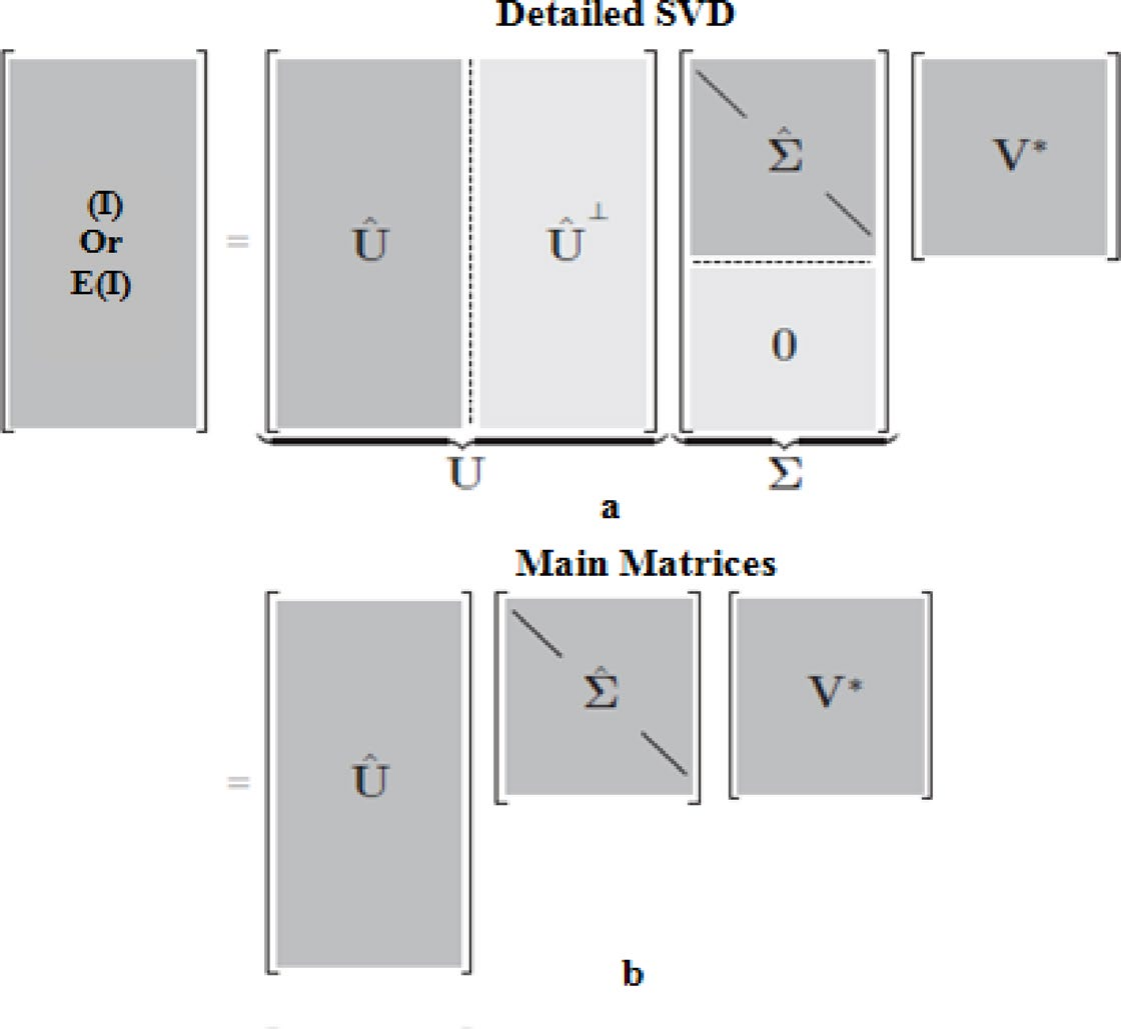
Schematic contents of the SVD matrices, (**a**) Detailed contents, (**b**) Main/Economic matrices^43^.

In general, the decomposition process using data transforms is a widely used in the reduction size of data, security-encryption in a different domains and data hiding techniques^39–43^. The SVD is a linear algebra technique, it is used in the process of a real or complex matrix/data factoring into three smaller matrices which are the side information (U and V matrices) and the diagonal matrix (singular values (SV). A useful SVD technique is to draw attention to the matrix’s attributes and factorize it into a series of smaller matrices having more definite qualities. Based on the properties of SVD decomposing technique, it adopted to establish loss-less data hiding steganographic approach through hiding the classified information/images into the SV matrix. The embedding process has been executed using the plaintext of the classified information or ciphered version after the encrypting process (As an optional in the proposed approach). Figure 1 shows the detailed contents of the SVD technique in the matrix decomposing and reduced matrices. Table 2 gives the description of the utilized symbols and notations in the analysis of the proposed security approach as clarified in the following section.

**Table 2:** The utilized Math symbol/notation in the presented analysis of the proposed Triple-layered security.

Symbol	Description
*I*	Image
*M*	Size of image pixels
M*M	Image dimension
*Conv*	Convert Image from color to gray
*I*_*E*_	Image after Encryption
*Enc*_*baker*_	Encryption by chaotic baker map
*C*	Cover Image
*SVD*	Singular Value Decomposition
*U and V*	The side information matrices
*∑*	The diagonal matrix
*K*	Multiplying factor
${I}_{E}{\prime}$	Encryption image after multiply a factor
${\sum }{\prime}$	The diagonal matrix after embedding an encryption image
*C’*	Stego image
*F(x,y)*	original image
*f(X,Y)*	marked image
*N*	Size of image pixels
*f(i,j)*	original image
${f}{\prime}\left(i.j\right)$	marked image
*i*	Row dimension of message
*J*	Column dimension of message
$\mu$	Mean of image pixels
$\sigma$	Variance of image pixels
*C*_*o1*_	Cross correlation

**Fig. 2:**
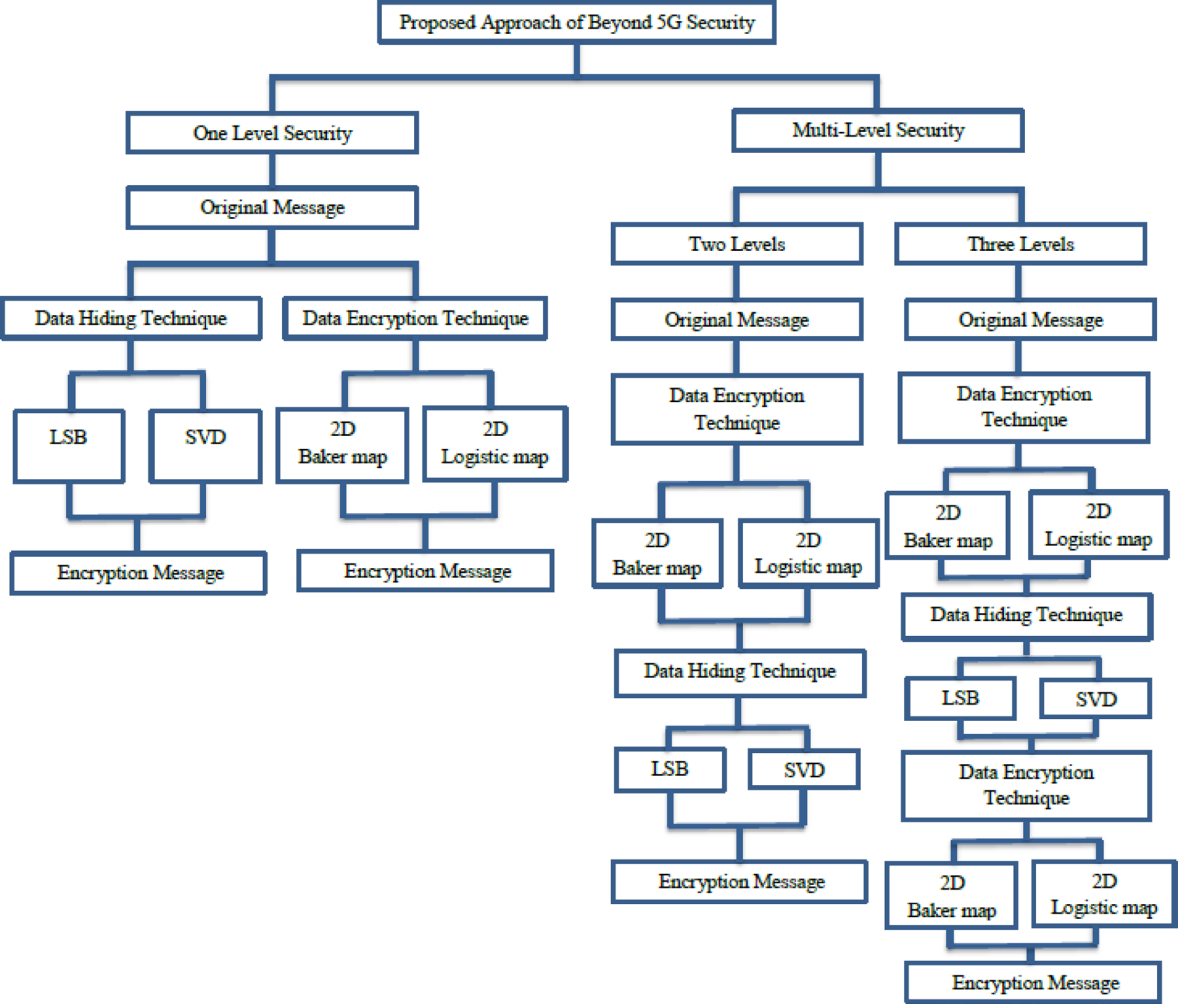
The proposed adaptive security approach for 5G/beyond networks and its various stages.

The second portion in the proposed approach is the chaos-cryptographic technique. This portion is utilized to enhance the robustness and adding flexibility in applying of the embedded security levels through applying baker map-based encryption tool ^44^. This chaos-based encryption has a high attacks/noise resistance and efficient in the key management. The second chaos mapping is the logistic map-based chaos encryption tool, it is utilized because its high key sensitivity and tampering detecting^45–47^. Due to these properties, the logistic map-based chaos encryption tool has been applied in the proposed approach in specific/last stages only or according to the conditions of transmission/storage medium^48–50^. The stages and available security layers have been clarified in Fig. 2.

### Methodology of triple-level security system

In this section, the proposed Triple-level security systems has been presented as an implemented example for the security of 5G/beyond vision. The proposed security system consists of three levels of data security. The security system suggests that the levels consist of two levels of data encryption techniques and one level of data hiding techniques, so that the original message passes through three levels of security. We present this proposal to provide a high level of security for data transmission over 5G/beyond network.

**Fig. 3:**
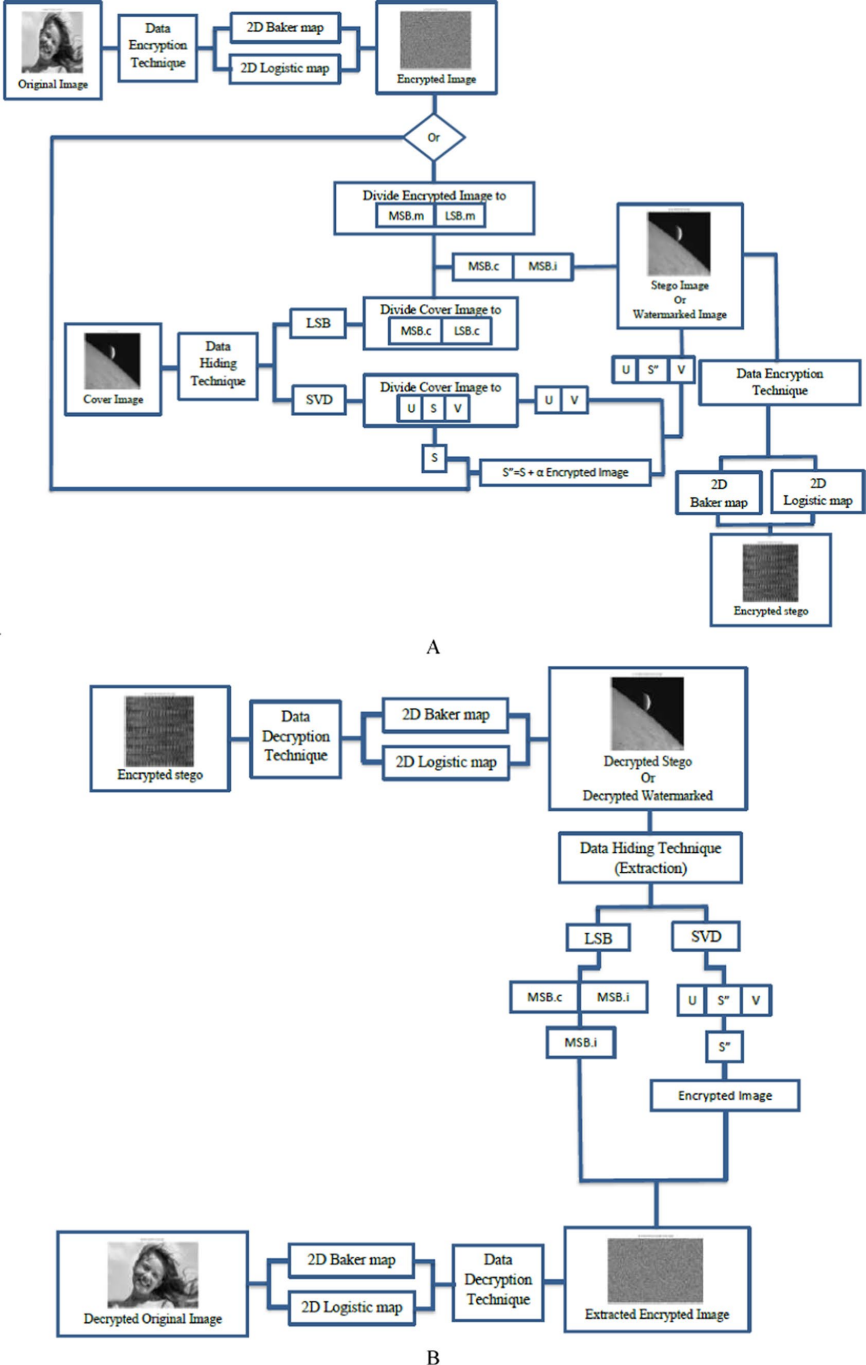
Contents and stages of the Triple-Level security system in the two stages of (**A**) Encryption/Embedding, (**B**) Extracting/Decryption.

**Fig. 4:**
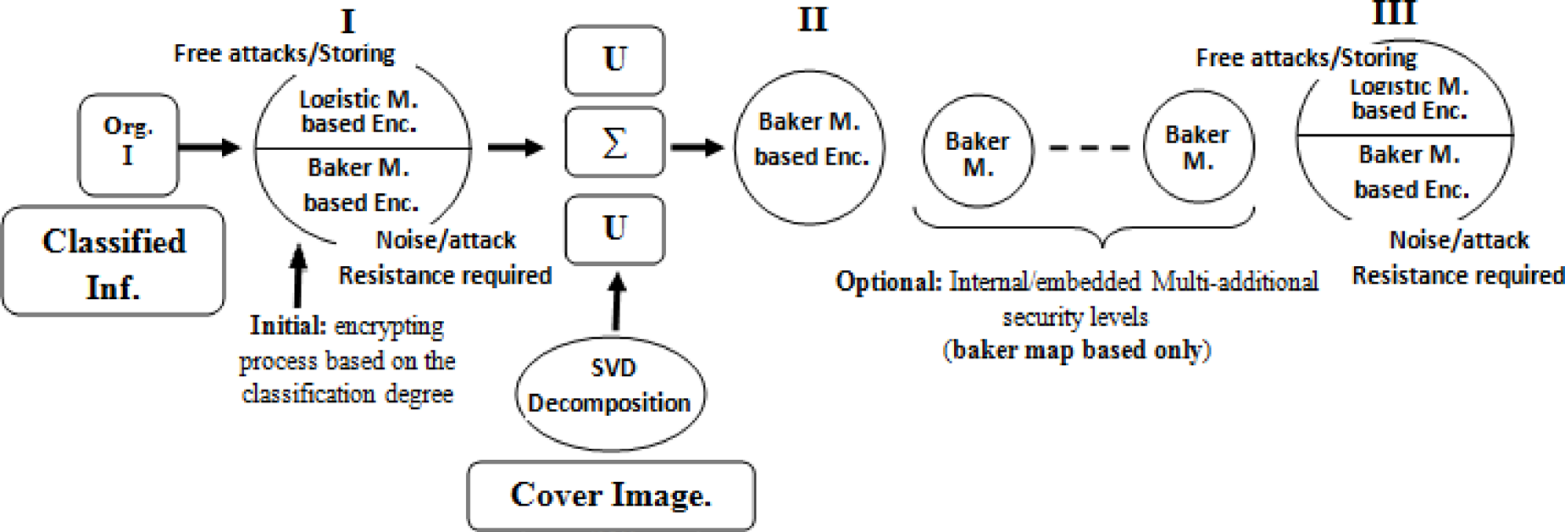
The Proposed our design detailed/steps of the Triple-level security approach.

**Fig. 5:**
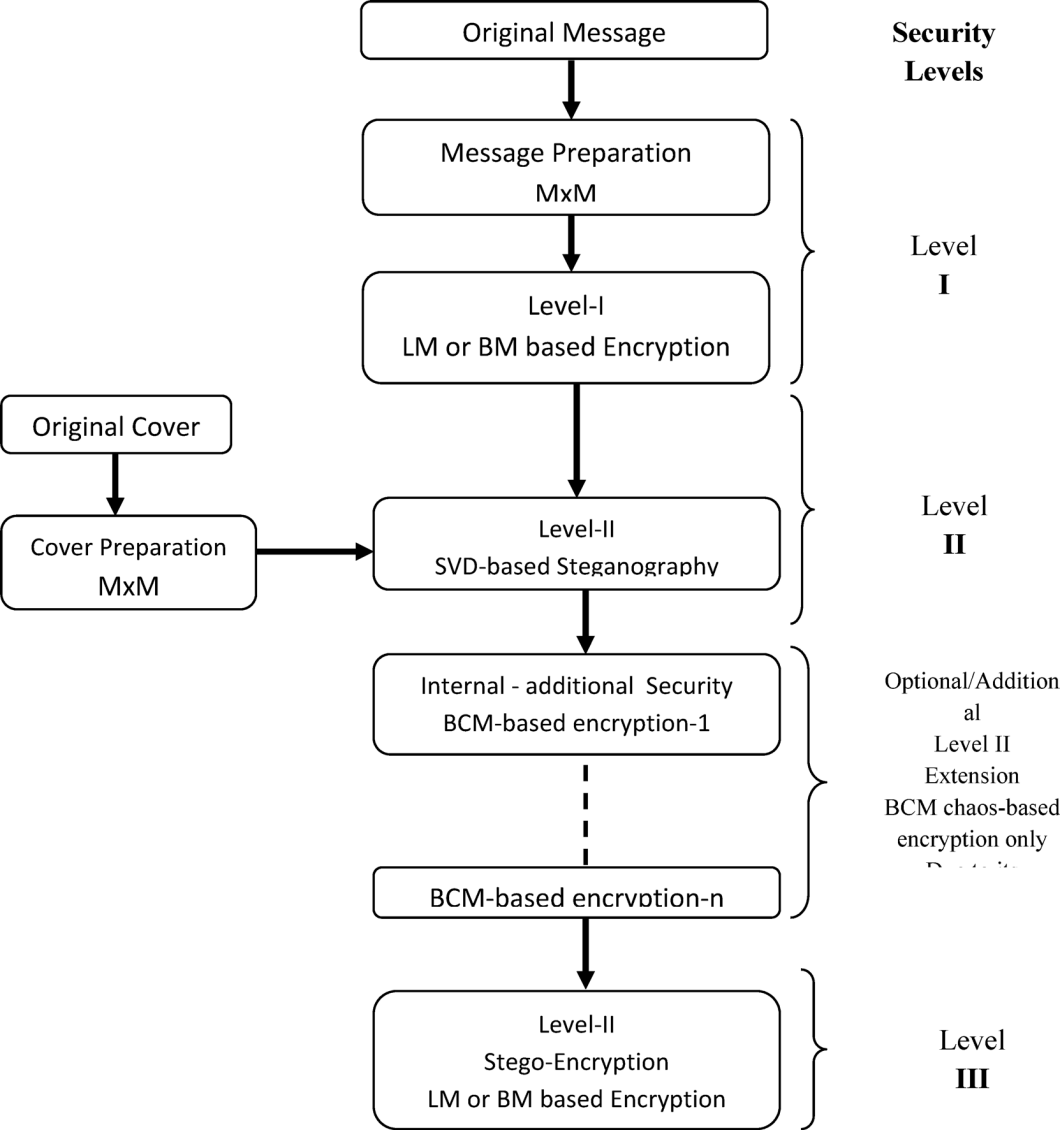
Layered-flow of the proposed Triple-level security approach.

Figure 3 shows the components of a Triple-level security system and demonstrates the steps of the original plaintext processing. The Triple-level system works by encrypting the original message using one of encryption technique as a first stage, then hiding the original message within another message using one of data hiding techniques as a second stage, and then encrypting the message again using one of encryption technique as a third stage.. In the evaluating section, the LSB-based data hiding steganographic has been ignored due to its losses, the SVD-based steganographic has been considered due to the results of preliminary experiments. These experiments revealed the transparency of SVD steganographic and high quality extracted mark compared to the LSB-based technique. The analysis and comparison will be considered in the future work as mentioned in section “Conclusion”. Our proposed design has been clarified in Fig. 4, it gives the detailed processes of the triple-level security approach with the unlimited number of embedded/internal additional security levels as required. Figure 5 clarifies the layered flow of the proposed approach.

In the following, the operation mechanism of the proposed adaptive Triple-level security approach has been listed a simple step-by-step sequences.

Step: 1 The classified information/image preparation: RBG (x, y, z) image conversion to gray scale within square dimensions.

$${\text{Original Gray }}\left( {I \, \left( {MxM} \right)} \right)\, = \,{\text{Conv }}\left( {{\text{RBG}}\left( {x, \, y, \, z} \right)} \right)$$

Step: 2 (I-level):The initial encrypting process using 2-D baker map-based chaos cryptographic tool.

$$I_{E} \, = \,{\text{Enc}}_{{{\text{bake}}r}} \left( {{\text{Gray}}(I\left( {MxM} \right)} \right)$$

Step: 3 The decomposition process of the cover image (C).

$${\text{SVD}}\left( C \right)\, = \,Ux\sum xV$$

The U and V are the side information matrices, the ∑ represents the diagonal matrix, which is used to embed the classified information/encrypted image $(I_{E} \, = \,{\text{Enc}}_{{{\text{baker}}}} \left( {{\text{Gray }}\left( {I \, \left( {MxM} \right)} \right)} \right)$ .

Step: 4 The weighting process: in this step, the attenuating of I_E_ has been performed by multiplying it in K factor. To maintaining the non-observable mark after the embedding process, K must be less than 0.1 (K = 0.01).

$$I^{\prime}_{E} = K*I_{E}$$

Step: 5 (II-level): The embedding process of attenuated encrypted image (${I}_{E}{\prime})$ into the diagonal matrix (∑) to produce the marked ∑ $\left( {\sum^{\prime}} \right)$.

$$\sum^{\prime} = I^{\prime}_{E} + \sum$$

Step: 6 Reconstruction the marked cover by inversing the process of decomposition to produce the stego signal, ( the embedded mark ‘encrypted information/image’ within the cover).

$$Inverse \, \left( {SVD} \right)C^{\prime } = U \times \mathop \sum \limits^{\prime } \times v$$

Step: 7 The optional number of embedded/internal security level as required using baker map-based chaos cryptographic tool due to its mentioned properties.

Step: 8 (III-level): the processes of encrypting the stego- signal which contains the encrypted mark, This stage has been performed to produce encrypted-stego signal $Enc\;\left( {C^{\prime}} \right)$ . The encryption process has been executed using one of the chaos-based cryptographic tool according to the medium and pre-determined conditions.

As clarified from these previous steps of encryption stage, the proposed main Triple-level security approach provides a security approach for 5G/beyond networks with the following features, the robustness, flexible complexity and adaptively. It successes in the proposed design to build research gap of the 5G/beyond security networks.

## The objective metrics: performance evaluations

The efficiency, reliability, applicability, and complexity measuring of the proposed Triple-Level Security System are performed based on evaluating quality of extracted/decrypted image.

This section defines the different performance metrics, which are used for evaluating the robustness and reliability of the proposed cascaded-interactive cryptographic algorithm depending on evaluating the time complexity, extracted image quality, decrypted image quality, attacks resistance and attack sensitivity. Several metrics tools have been employed for measuring the similarities of original image and extracted/decrypted image. Also, the efficiency of the presented crypto-stego algorithm is evaluated through evaluating the classified image quality. The used metrics is described as follows ^51–55^:-

- Correlation Coefficient (Cr):

It is one of the most common used metrics to evaluate the degree of closeness between the two functions. This metric can be used to determine the extent to which two images are close to each other, as given in Eq. (1). f(X, Y) represents the extracted message and F(x, y) denotes the original message.

1 $$\text{Cr}=\text{ Corr}(\text{F}(\text{x},\text{ y}),\text{ f}(\text{X},\text{ Y})$$

So, it gives a direct measure of the proposed algorithm efficiency regarding to the quality of the extracted classified message. The most efficient algorithms produce images with correlation ratios closer to unity.

- Mean square error (MSE):

MSE is one of the most important image quality evaluation metrics, and it can be defined as the average of the squares of the difference between the intensities of two examined images. It can be mathematically represented as in Eq. (2),

2 $${\text{MSE}} = \frac{1}{{{\text{MN}}}}\mathop \sum \limits_{i = 1}^{M} \mathop \sum \limits_{j = 1}^{N} \left( {f\left( {i,j} \right) - f^{\prime}\left( {i.j} \right)} \right)^{2}$$

where f (i, j) is the original image and f’ (i, j) is the marked image. Higher values of MSE mean that the image has a poor quality.

- Peak Signal-to-Noise Ratio (PSNR):

The third utilized metric is the PSNR, The PSNR can be formulated mathematically as in Eq. (3),

3 $$\text{PSNR }\left(\text{dB}\right)=10\text{log}\left(\frac{{255}^{2}}{\text{MSE}}\right)$$

A higher value of PSNR is better, where it means high quality of extracted message.

- Structural similarity (SSIM):

The SSIM is a recently proposed to measure the image fidelity, it has proved highly effective in measuring of the signals fidelity. The human visual system is highly sensitive to structural distortions and easily compensates for nonstructural distortions. The main function of the SSIM is to simulate this functionality, it is calculated as follows:-

Let x = {xi|i = 1, 2, …,N} and y = {yi|i = 1, 2, …,N} represent the original and the test image signals, respectively. Then, the SSIM can be expressed as in Eq. (4):-

4 $$\text{SSIM }\left(\text{x},\text{y}\right)= \frac{\left(2{\upmu }_{\text{x}}{\upmu }_{\text{y}}+ {\text{C}}_{\text{o}1}\right)(2{\upsigma }_{\text{xy}}+ {\text{C}}_{\text{o}2})}{\left({\upmu }_{\text{x}}^{2}+ {\upmu }_{\text{y}}^{2}+{\text{C}}_{\text{o}1}\right)({\upsigma }_{\text{x}}^{2}+ {\upsigma }_{\text{y}}^{2}+ {\text{C}}_{\text{o}2})}$$

## Computer simulation and result discussion

In this section, we present a collection of various simulation experiments, to illustrate the best performance of a Triple-level security system. These experiments demonstrate the results of combining different data hiding techniques and various data encryption techniques within the different merging scenarios. They have been implemented using LSB and SVD techniques as data hiding techniques, while 2D-Baker Map and 2D-Logistic Map techniques have been employed as a data encryption techniques. These techniques have been combined in a different arrangements to determine the robust and reliable performance of the proposed approach. The simulation and modeling are executed using Windows 2010 and MATLAB version 2014.

**Fig. 6:**
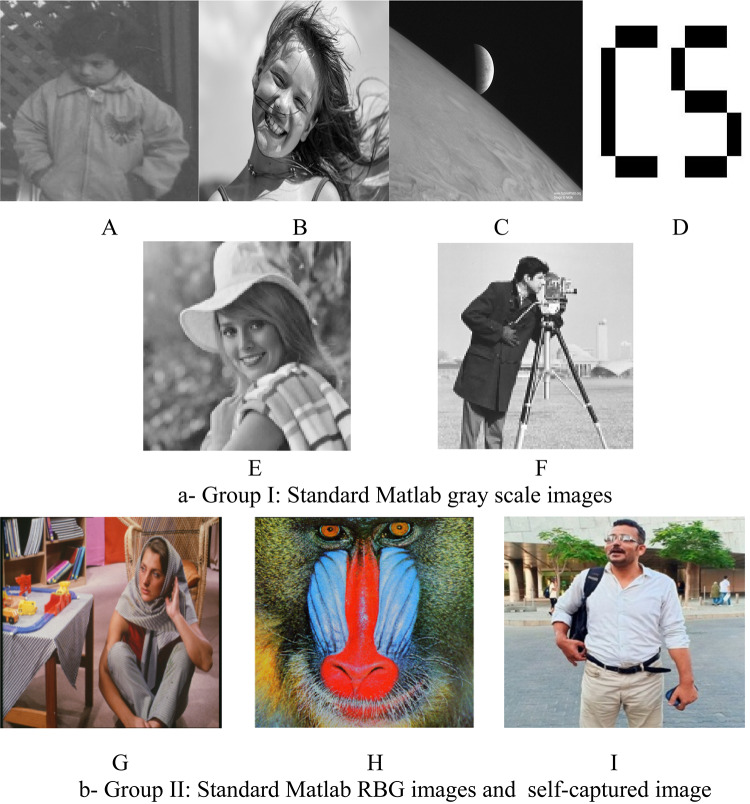
The utilized Matlab images for the computer simulation experiments, (**a**)- Group I of gray scale images, (**b**)- RBG images. *Notes*: All the utilized images in this research paper are standard Matlab images except our self captured photo (No. I).

The simulation experiments utilize a set of the different standard Matlab images, both in terms of size and black-and-white colors. The results analysis demonstrate and evaluate the performance of proposed approach with respect to the use of a variety of image quality measurement tools. The used images in the computer simulation experiments are displayed in Fig. 6.

### Triple-level security system (2D Logistic map&SVD&2DBaker map) encryption techniques

In this section, we discuss simulation experiments for using merge between 2D Logistic map, SVD and 2D baker map techniques as a triple-level of security. The simulations demonstrate the robustness and reliability of this scenario in data security and its impact on the secured data. The simulations also determine the loss due to the proposed approach or there is not. To ensure the quality and robustness of the approach with respect to the image size, the experiments have been implemented using a set of different images with varying sizes (256*256 & 512*512 & 1024*1024) and to verify the credibility of the simulation results.

**Fig. 7:**
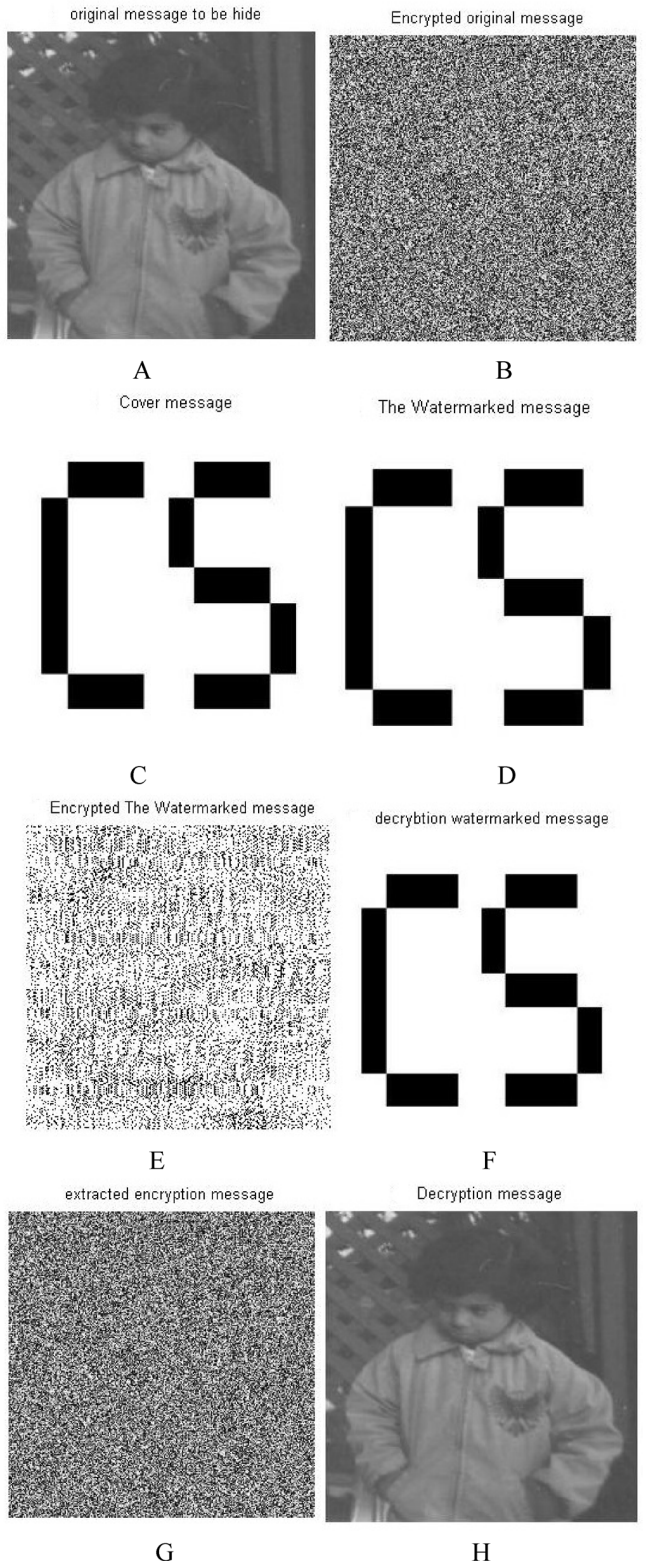
The standard image of Matlab using Triple-level security system (2D Logistic map &SVD&2DBaker map) technique, (**A**) Original message, (**B**) Encrypted original message, (**C**) cover message, (**D**) watermarked message, (**E**) Encrypted watermarked message, (**F**) decrypted watermarked message, (**G**) extracted watermarked message, (**H**) decrypted original message. *Notes*: All the utilized images in this research paper are standard Matlab images except our self captured photo (No. I).

Figure 7 shows the results sample of images with the size 256*256 . Simulation experiments have been carried out using two different images, one of images is used as a cover message, while the other image is used as the original message. By applying the proposed approach, the original message has been encrypted using the 2D-Logistic map encryption technique, which is the first stage of the security system. The encrypted original message has been hidden inside a cover message using SVD, it is the second stage of the security system. In the next stage, the watermarked message has been encrypted using the 2D-baker map encryption technique, it is the third stage of the security system. After applying the three stages, we have a triple-level of security for providing a robust and reliable secure message. The encrypted watermarked message has been produced after executing the third stage. To decrypt the secure message, the Triple-level security system is reversed from the third stage to the first stage. The encrypted watermarked message is decrypted using 2D baker map decryption to decrypted watermarked message, then the decrypted watermarked message is decomposed using SVD tool to the extracted watermarked message with respect to the (K = 0.01 attenuating factor) and finally, the extracted encrypted watermarked message is decrypted using 2D-Logistic decryption to the decrypted original message.

Through the application of a set of image quality tools, a comparing between the original message and the decrypted message to measure the similarity. These comparison has been performed to determine the effect of the encryption/decryption algorithms on secured data and determines its reliability and robustness based on the quality of the extracted message. Figure 7 shows the match between images before and after applying the Triple-level security system. The match appears between the original message and the decrypted message, as well as between the encrypted original message and extracted watermarked message, as well as between the cover message, watermarked message and decrypted watermarked message. This figure indicates that encryption techniques/SVD did not affect the messages, where there is no loss due to the proposed security approach. These match appeared in the results of the image quality tools, which are shown in Table 3.

**Table 3:** Metrics of the image quality Triple level security system (2D Logistic map&SVD&2DBaker map).

Image quality metrics		256-A	256-B	256-C	512-A	512-B	512-C	1024-A	1024-B	1024-C
Corr original message and decryption message	1		1	1	1	1	1	1	1	1
PSNR	99		99	99	99	99	99	99	99	99
MSE	0		0	0	0	0	0	0	0	0
SSIM original message and decryption message	1		1	1	1	1	1	1	1	1

In the devoted experiments, two group of images have been utilized to measure the reliability of the proposed security approach and its suitability/applicability for the gray scale and RBG images. All the used images from the Matlab S/W package except the image No. I, it is captured by our self.

As clarified from the preliminary simulation experiments, our proposed security system produces high quality extracted/decrypted images after 2-encryption processes and the embedding process. The multi-embedded cryptography stages can be performed based on the chaos-mapping technique to achieve additional/internal security levels with the variations in the chaos-maps keys to enhance the confidentiality of the proposed approach.

**Fig. 8:**
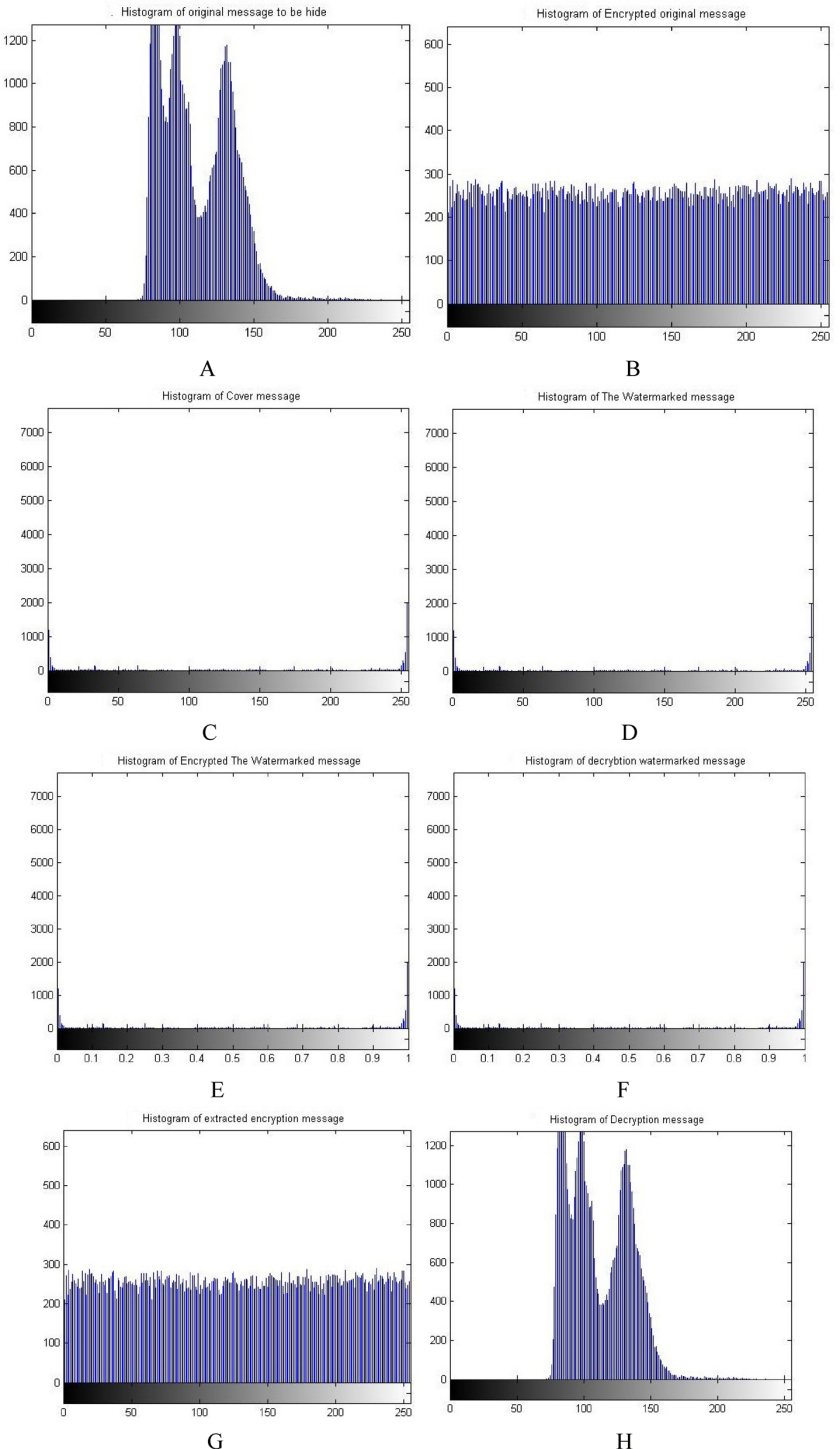
Histogram of The standard image of Matlab using Triple-level security system (2D Logistic map &SVD&2DBaker map) technique, (**A**) Original message, (**B**) Encrypted original message, (**C**) cover message, (**D**) watermarked message, (**E**) Encrypted watermarked message, (**F**) decrypted watermarked message, (**G**) extracted watermarked message, (**H**) decrypted original message.

Figure 8 shows the histograms of the images which have been shown in Fig. 7. The histogram of the images indicates match between images. It is observed that the match between the histogram of the original message and the histogram of the decrypted message, as well as a match between the histogram of the encrypted message and the histogram of the extracted watermarked message. Also it has been observed that the match between the histogram of the cover message, the histogram of the watermarked message and decrypted watermarked message. This result indicates that encryption techniques and SVD do not affect the messages and there is no loss. These matches demonstrate the robustness and reliability of proposed security approach.

**Fig. 9:**
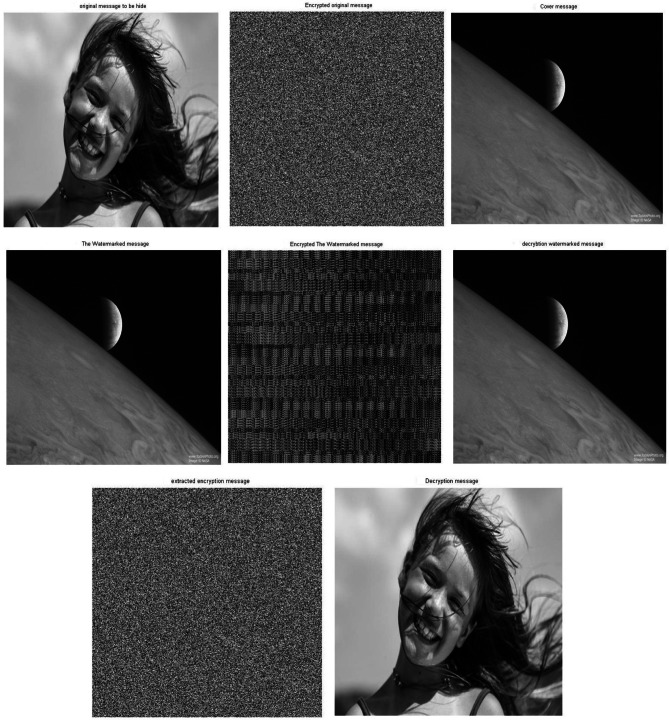
Images sample of the original and decryption image using Triple-level security system (2D Logistic map &SVD&2DBaker map) technique size 512*512.

**Fig. 10:**
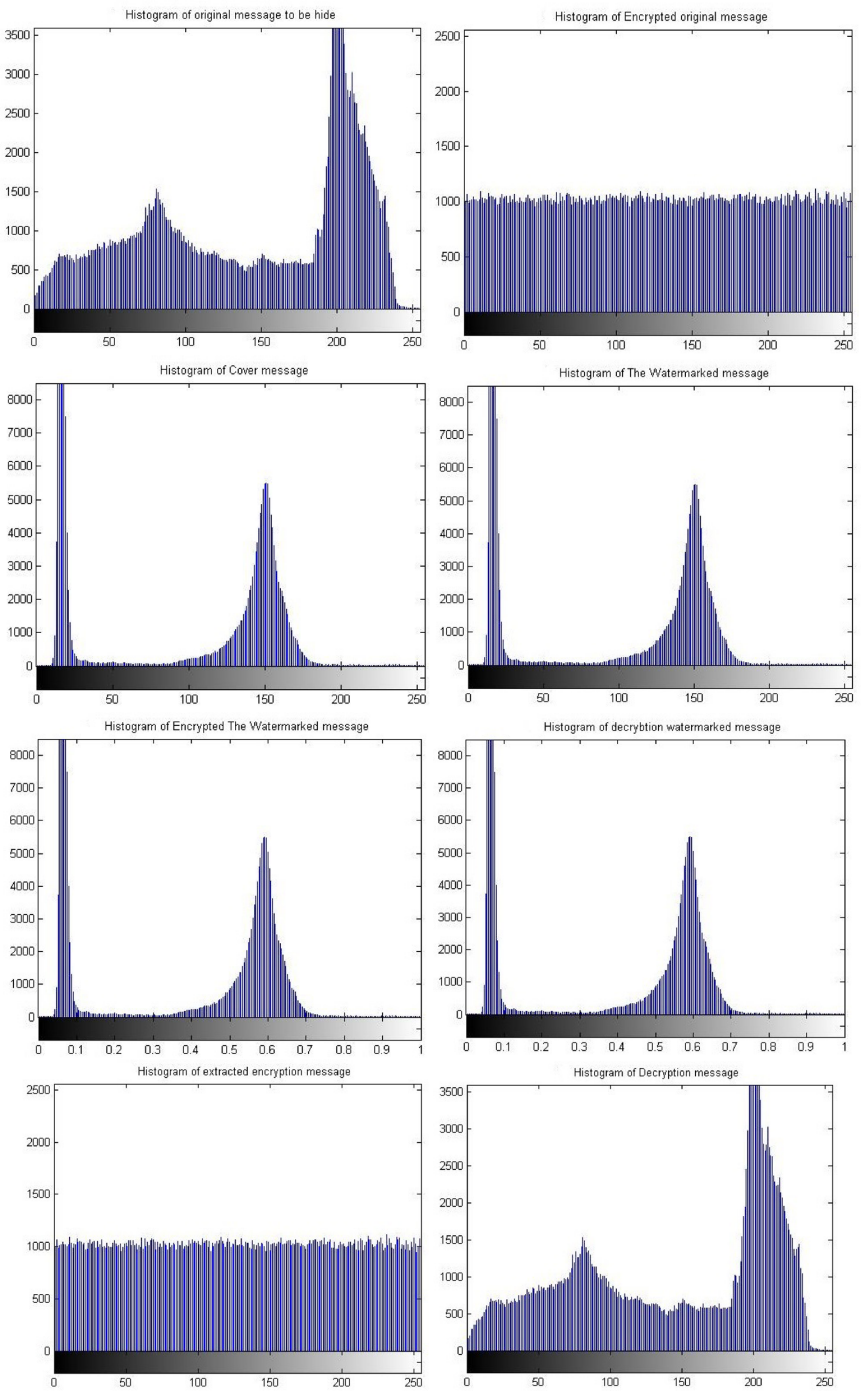
Histogram of images sample of the original and decryption image using Triple-level security system (2D Logistic map &SVD&2DBaker map) technique size 512*512.

**Fig. 11:**
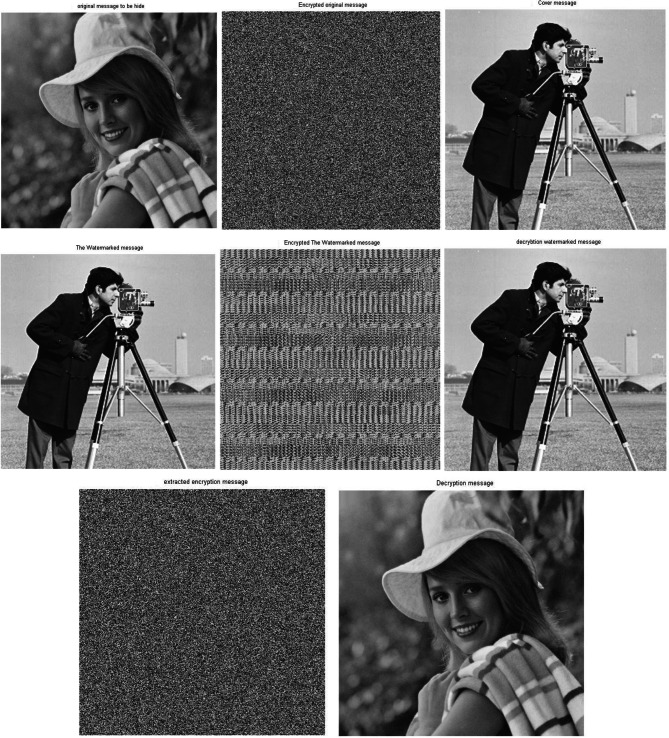
Images sample of the original and decryption image using Triple-level security system (2D Logistic map &SVD&2DBaker map) technique size 1024*1024.

**Fig. 12:**
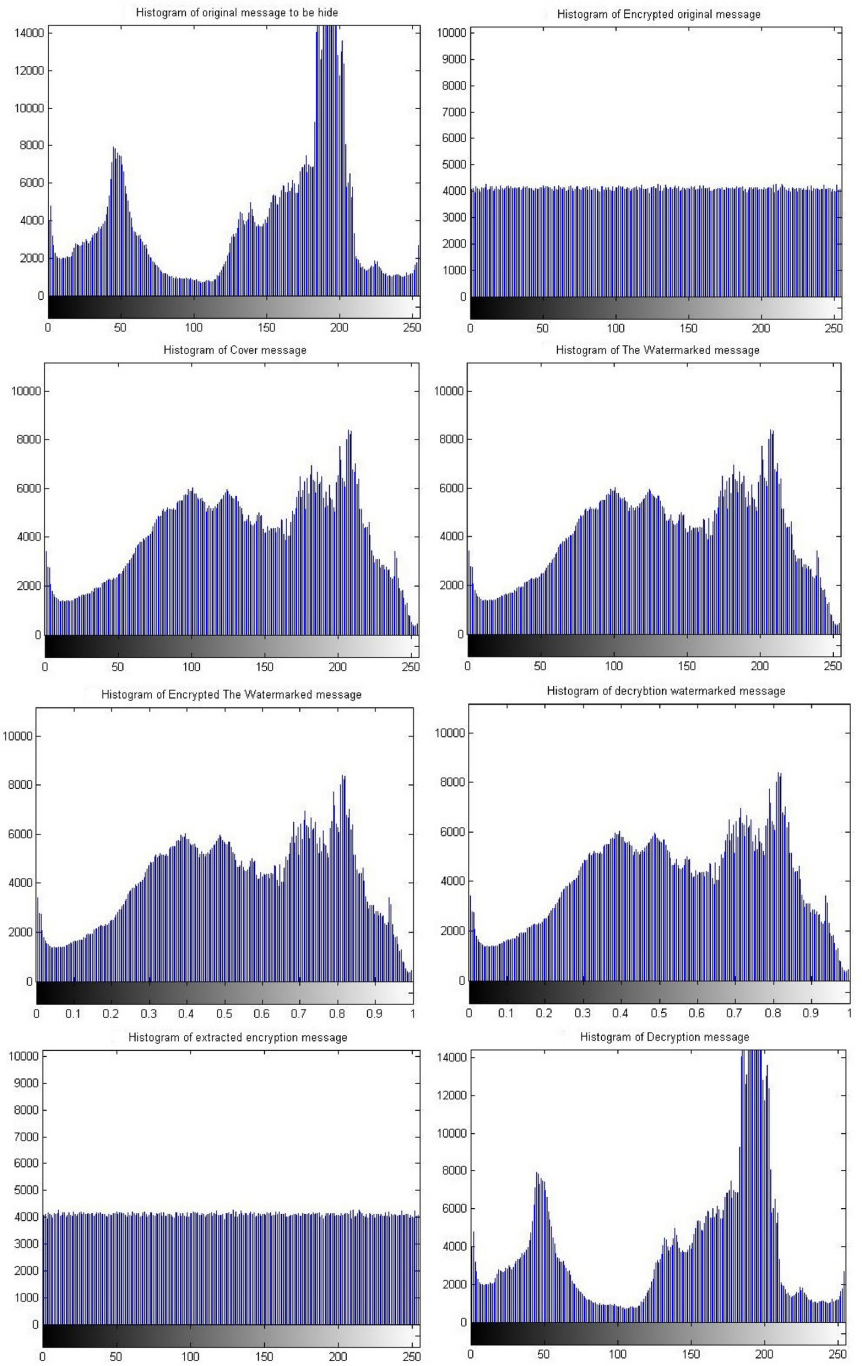
Histogram of images sample of the original and decryption image using Triple-level security system (2D Logistic map &SVD&2DBaker map) technique size 1024*1024.

Simulation experiments have been carried out again on a different set of images with sizes 512 * 512 and 1024 * 1024 using the same scenario of the proposed security approach. This group of experiments utilize different sizes of images to measure the performance of the proposed approach in the case large amounts of data processing. The simulation results indicated no significant effect due to the different size of the images. However, the results indicate the same matches that appeared with the results of the images of size 256 * 256. Consequently, there is a match between the original message and the decrypted message. These results have been shown in Figs. 9 and 10 as well as Table 3, for images of size 512 * 512. However, Figs. 11 and 12, as well as Table 3, shows results for images of size 1024 * 1024.

Table 3 shows the results of simulation experiments using a set of different images using the scenario of (2D-Logistic map&SVD&2D-Baker map) security technique. The table shows the results of the image quality measurement tools used in the simulation experiments. The results indicate the presence of match between the original message and decrypted/extracted message. Therefore, the results indicate that encryption techniques and SVD did not affect the messages and there is no loss due to the presented approach or the various size of processed message. These matches demonstrate the robustness and reliability of proposed security approach ‘Triple- level security system’ for providing secured message .

**Fig. 13:**
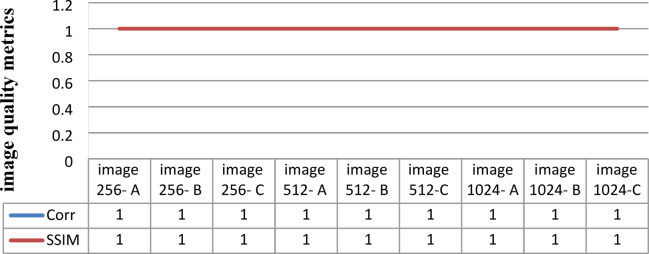
The result of Cr& SSIM between original message & extracted message.

Figure 13 shows the results of correlation coefficient (Cr) and Structural Similarity Index Metric (SSIM) between the original image and the decrypted/extracted image. In Fig. 13, the curves show that the values of Cr and SSIM are equal 1; it indicates a complete 100% match between the original message and the decrypted message. The curves also show that the difference in image size did not have an effect on the match between the images. The curves indicate that Triple-level security system in this form/scenario has not affected the extracted images. It also shows that the images have not loss due to the processes of cryptographic or data-hiding tools..

### Triple-level security performance with the RBG images

**Fig. 14:**
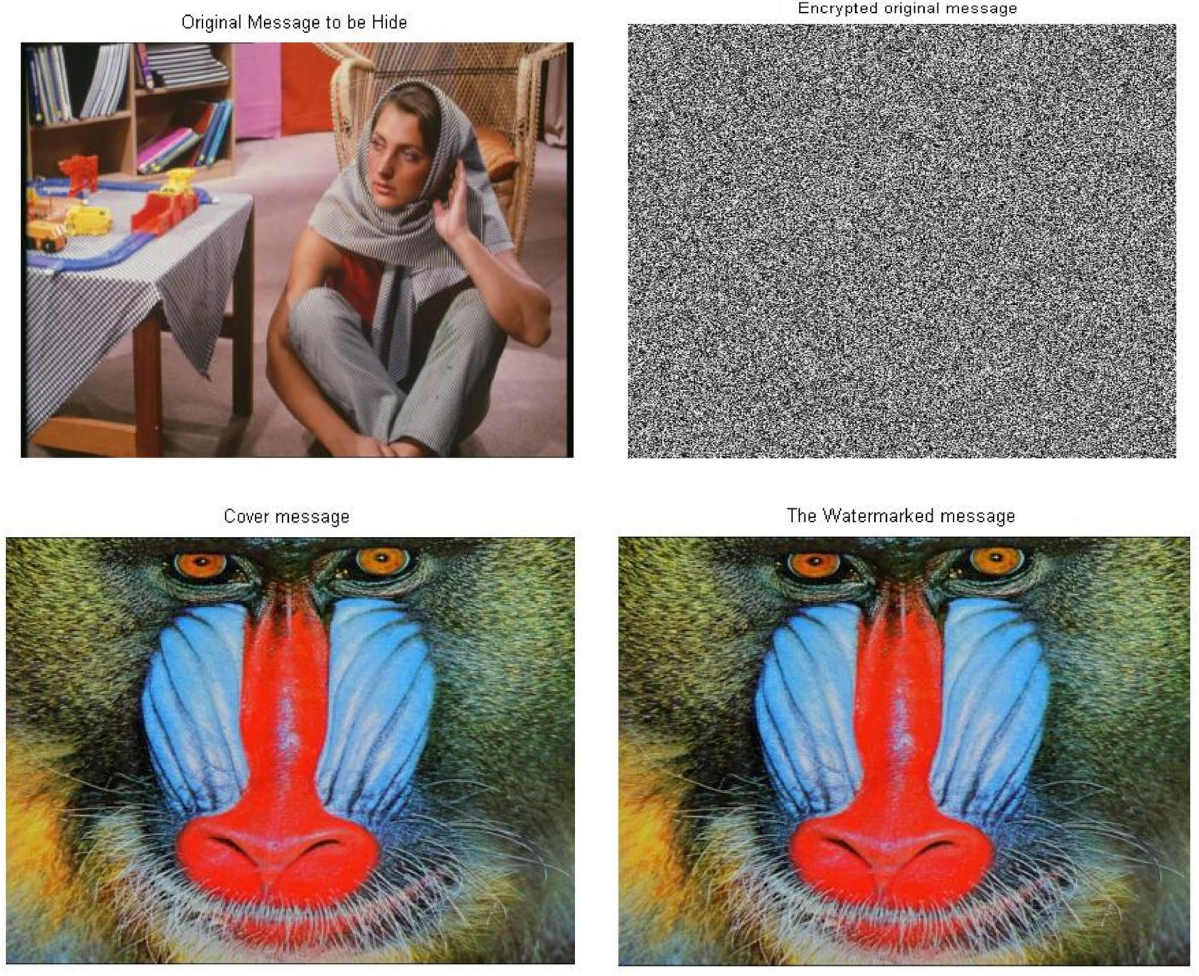
Images samples of RBG image security using Triple-level security approach original Woman image, encrypted Woman Image, original cover and stego-after embedded(SVD&2DBaker map) technique size 300*400*3

This section has been demonstrated to evaluate the applicability of the proposed security approach for securing the RBG images. As mentioned in section “The proposed security approach of 5G/beyond networks”, RGB images requires additional step in the preparation stage, this process of RBG preparing has been considered in the simulation modeling. As shown in Fig. 14, the mark ‘classified information/image’ is no-observable completely, it has a high quality due to the loss-less embedding process and transparency of utilized cryptographic- chaos based tools. The standard colored Woman image has been used to test the performance of the proposed approach.

**Fig. 15:**
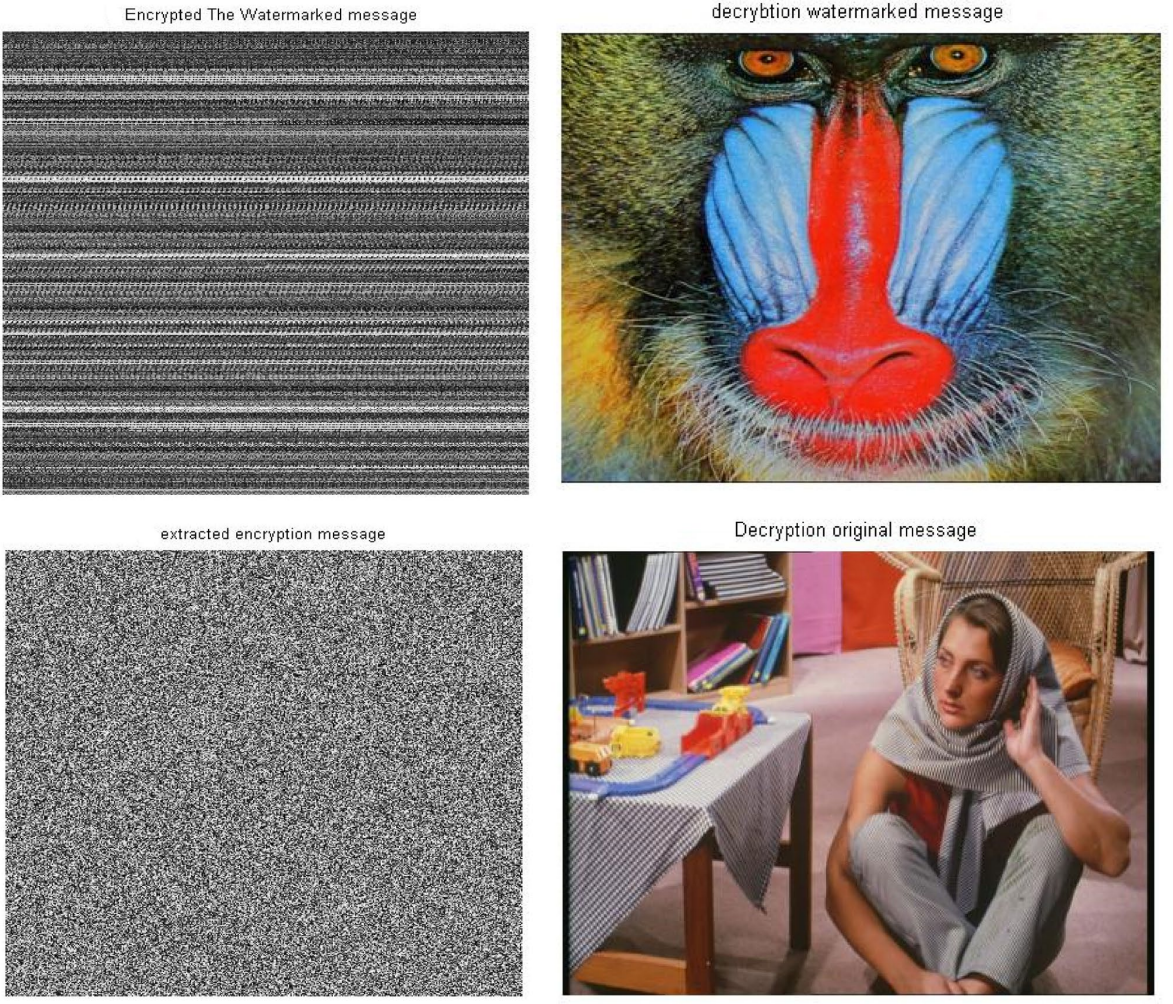
Images samples of extracted process of Woman RBG image using Triple-level security approach, Encrypted stego, decrypted stego, and decrypted Woman Image, extracted/decrypted Woman mark(SVD&2DBaker map) technique size 300*400*3. *Notes*: All the utilized images in this research paper are standard Matlab images except our self captured photo (No. I).

The efficiency of the extraction process of the proposed approach has been evaluated through showing the samples of encrypted stego image, the decrypted stego, encrypted Woman image and the extracted/decrypted Woman image as given in Fig. 15. The results clarify the robustness of extracting process, the transparency of the proposed security approach due to the high quality of the classified Woman image after the extraction process. On the other hand, the cover is not impacted due to the attenuating process of the mark image as mentioned in the description of embedding steps (K < 0.1).

**Fig. 16:**
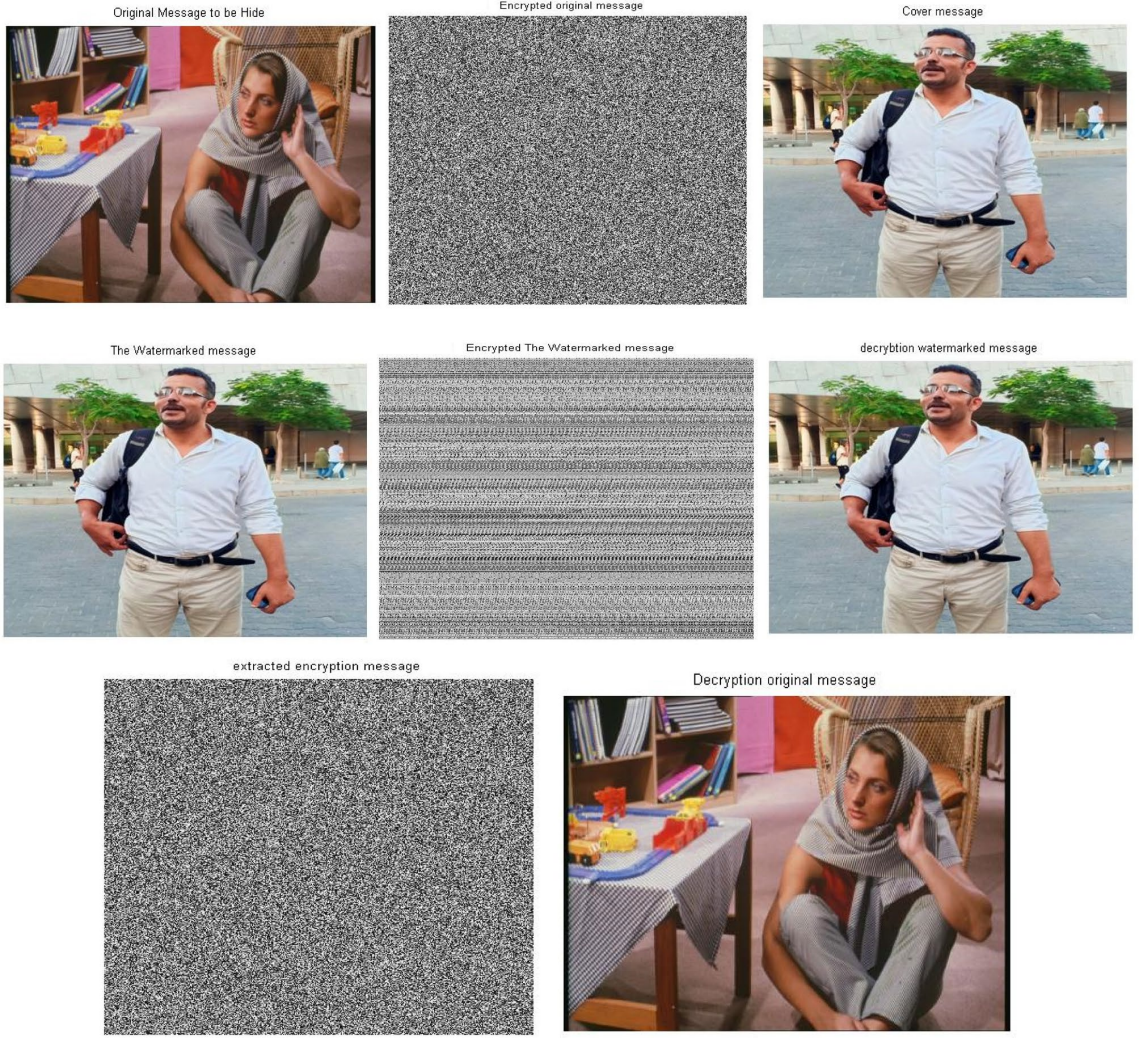
Images samples of extraction process of Woman RBG image using Triple-level security approach, (**a**)-Original Woman Image, (**b**)-Encrypted Woman Image , (**c**)-Original Cover, (**d**)-The watermarked message ‘Stego’, (**e**)-Encrypted Watermarked message ‘Stego’, (**f**)- Decrypted Watermarked message ‘Stego’, (**g**)-Extracted/encrypted Woman Image, (**h**)- Extracted/decrypted Woman image (Used self captured image No. I).

For testing and evaluating the applicability of the proposed security approach and its suitability for RBG images securing over the 5G/beyond networks, the previous experiments has been repeated using a different RBG images. The results of the colored Woman image experiment have been shown in Fig. 16 using un-standard colored image as a cover. As clarified from these results and the images samples which are produced through executing the experiments, the extraction process proves the similarity of decrypted/extracted Woman image 100% with the original version ‘Plaintext’. The proposed security approach has a triple-level of security, the cryptographic-chaos based is utilized to encrypt the classified plaintext, the embedding process using the loss-less SVD-based steganographic tool and in the last level the cryptographic-chaos based to produce the ciphering watermarked message.

Several experiments have been devoted to evaluate the performance of the proposed Triple-level security approach. These experiments have proved the superiority and high transparency of the proposed approach compared to the existing and related techniques. The additional feature in the proposed approach is the flexibility in the security level executing, Triple-level, Two-level, one-level and none. The internal/embedded rounds of cryptography processes on the already encrypted watermarked message, these internal stages are optional based on the nature of classified information or the conditions of transmission.

The results of extracted colored woman image have been tabulated in Table 4, the tabulated result clarifies that the quality of extracted/classified watermark woman image, it has the 100% similar the original plaintext. Also, the results prove the non-observable mark, where the watermarked message is 100% similar the original cover image due to the values of metrics. The similarity of watermarked message and the original mark is achieved by choosing a true attenuating factor (K) for the classified ‘mark’ message (*K* = 0.01).

**Table 4:** Metrics of the Watermarked message and colored Woman image quality using Triple level security system (2D Logistic map&SVD&2DBaker map) with respect to the various metrics.

Image quality metrics	Cover & Stego messages	Original & Decryption messages
Cr	1	1
SSIM	1	1
PSNR	99	99
MSE	0	0

### Comparison the proposed approach & related works

This section has been devoted to clarify the robustness of the proposed security approach and its superior through the comparison with the recent published related works. The presented comprehensive comparison involves the quality of classified/extracted message ‘embedded mark’ and the watermarked message with respect to the utilized metrics for these purposes in each related research work separately. Table 5 gives tabulation of the comparison of the Triple-level security approach with the recent related works of the 5G/beyond security approaches.

**Table 5:** Comparison of the proposed approach with the related works with respect to the various metrics of the ‘transparency’ and the classified information quality.

Refs	Transparency “Cover & Stego message”				Original & decryption message			
	Cr	SSIM	PSNR	MSE	Cr	SSIM	PSNR	MSE
Ref. ^17^	–	–	–	–	–	0.9337	33.8483	–
Ref. ^18^	–	–	65.67	–	–	–	–	–
Ref. ^27^	–	0.9769	41.3706	–	–	–	–	–
Ref.^28^	–	0.9485	38.4167	9.3632	–	0.9485	38.4167	–
Ref.^29^	–	0.9803	42.8228	–	–	0.9855	42.8674	–
Ref.^30^	–	0.9999	48.1308	0.9999	–	1.0000	35.9995	16.3366
Ref.^31^	–	–	–	–	0.9996	–	31.2040	–
Ref.^33^	–	0.8633	42.63	0.4271	–	–	–	–
Ref.^34^	–	0.9983	42.6229	–	–	–	–	–
Ref.^35^	–	–	–	–	0.70778	–	18.0444	0.20658
The proposed triple-level approach	1	1	99	0	1	1	99	0

As shown from the tabulated values, the most of related research works have not utilize the sufficient metrics to measure the quality of the watermarked message, which is ‘denoted by the transparency’. The transparency means the effects of embedding process on the view of the original cover (The embedded mark is observable or not). Also, the quality of classified information ‘embedded mark’’ has been measured in the most of papers by insufficient metrics. On the other hand, the values of the metrics clarify the superior performance of the proposed Triple-level security approach. Hence, it successes to build research gap in the presenting security approaches of 5G/beyond network through the proposed design of the adaptive security approach.

### The complexity analysis

In this section, the complexity of the proposed approach has been considered and compared with the related works. As clarified from the results, the proposed approach has lower complexity due to the time of ciphering/hiding (2.13s) and time of deciphering/extracting (1.57s). The comparison of the performance of the proposed approach with the recent related works and existing techniques with respect to the complexity and methodology has been tabulated in Table 6.

**Table 6:** Comparison of the proposed approach with the related works with respect to the complexity.

Ref	Encryption time	Decryption time	Methodology/utilized techniques	Complexity degree	Security levels
Ref. ^21^	3.590	1.001	Synchronized chaotic system	High/Low	One/simple
Ref. ^25^	4	4	Hybrid encryption algorithm	High/High	Multi/complex
Ref. ^28^	2.2117	1.4732	A blind watermarking algorithm utilizing fast quaternion Schur decomposition	Low/Low	Multi/simple
Ref. ^31^	2.420631	1.911237	Multiple chaotic maps, watermarking and Arnoldscrambling algorithm	Low/Low	Multi/complex
Ref. ^34^	6.5812	2.4271	Chaotic and deoxyribonucleic acid encryption	High/low	One/complex
Ref. ^56^	15.8617	13.3824	Piecewise Linear Chaotic Map (PWLCM) , Logistic Map and DNA	High/High	Multi/complex
Ref. ^57^	12	11	EKbNV-SDT-AC Model	High/High	Multi/complex
Ref. ^58^	3.8	2.3	PSKAC	High/Low	One/complex
Ref. ^59^	2.5	1.71	ECC + AES	High/Low	Multi/complex
Ref. ^60^	3.0019	-	A combination of multiple chaotic maps	High/High	Multi/complex
Our proposed	2.1379	1.5717	Triple-level security approach	Low/Low	Multi/complex

The time complexity can be considered a real reflect of the computational complexity (CC), where the CC is the more general expression and related to the number of various operations for performing the algorithms. While the time complexity is defined as the time which is taken for running the algorithm, Hence, the CC is proportional to the time complexity, with increasing the number of operation, the running time increases ^61,62^. Therefore, the proposed approach improves the time complexity compared to the recent related published papers, and more suitable for the real-time applications.

We argue in this research paper the adaptation methodology in our proposed security approach, which has been adopted to build the research gap of adaptability of the existing techniques, that is lacking in some-way. The existing and related works missed a certain adaptation perspective. The flexible computation due to the boosting-gradient algorithm methodology has been adopted in the design of our proposed approach. These points are lacking in the existing techniques and related research works.

## Conclusion

This paper focused on the security of 5G/beyond networks. The paper presents the proposed example of the adaptation vision of 5G/beyond security. It introduces a proposed Triple-level security system to ensure effective data security on the network in adaptive behavior. Several simulation experiments have been performed to achieve the superior security scenario with the highest level of reliability and credibility. In the simulation experiments, encryption and data hiding techniques have been integrated in different ways to achieve adaptive and flexible security approach. The results indicate that the scenario of a Triple-level system consisting of 2D-logistic map, SVD, and Baker map, respectively performs better due to the transparency and time complexity. Image measurement tools indicated a perfect match between the original message and the decrypted/extracted message.. The results also indicate that there is no loss due to the proposed approach in the original message and that it was identical to the decryption original message, thus the system had no effect on the extracted message. Through these results, it is confirmed that the proposed Triple-level security is suitable and applicable for securing the transferred messages for a different real-time applications, due to its high reliability and credibility.. In future work, we will work on implementing the proposed Triple-level security system (2D logistic map & SVD & Baker map) for the different mobile wireless communication channels. Simulation experiments will be carried out to test the security system on a mobile wireless communication channel. These experiments will measure the robustness and reliability of the Triple-level security system in securing data in the presence of a noisy environment such as mobile wireless communication channel.

## Data Availability

The datasets used and/or analyzed during the current study available from the corresponding author on reasonable request. All the used images dataset from a standard Matlab images except one self captured image (No. I). Mathwork standard https://www.mathworks.com/help/images/image-import-and-export.html
